# An Integrative Framework of Stress, Attention, and Visuomotor Performance

**DOI:** 10.3389/fpsyg.2016.01671

**Published:** 2016-11-01

**Authors:** Samuel J. Vine, Lee J. Moore, Mark R. Wilson

**Affiliations:** ^1^Sport and Health Sciences, College of Life and Environmental Sciences, University of ExeterExeter, UK; ^2^School of Sport and Exercise, University of GloucestershireGloucester, UK

**Keywords:** stress, challenge, threat, anxiety, visuomotor control, performance

## Abstract

The aim of this article is to present an integrative conceptual framework that depicts the effect of acute stress on the performance of visually guided motor skills. We draw upon seminal theories highlighting the importance of subjective interpretations of stress on subsequent performance and outline how models of disrupted attentional control might explain this effect through impairments in visuomotor control. We first synthesize and critically discuss empirical support for theories examining these relationships in isolation. We then outline our integrative framework that seeks to provide a more complete picture of the interacting influences of stress responses (challenge and threat) and attention in explaining how elevated stress may lead to different visuomotor performance outcomes. We propose a number of mechanisms that explain why evaluations of stress are related to attentional control, and highlight the emotion of anxiety as the most likely candidate to explain why negative reactions to stress lead to disrupted attention and poor visuomotor skill performance. Finally, we propose a number of feedback loops that explain why stress responses are often self-perpetuating, as well as a number of proposed interventions that are designed to help improve or maintain performance in real world performance environments (e.g., sport, surgery, military, and aviation).

This article will review and draw together seminal theoretical explanations of performance variability under stress and argue for an integrative conceptual framework relevant to visually guided motor skill (visuomotor) performance (**Figure 1**). Specifically, in describing how individuals first respond to a stressful stimulus, we draw upon the work of Lazarus and Folkman (1984) [Cognitive Appraisal Theory (CAT)], and Blascovich (2008) (Biopsychosocial Model of Challenge and Threat, BPSM). While the aforementioned accounts of stress and human performance provide a detailed explanation of how the stress evaluation process may influence psychological and physiological reactions, they do not make specific predictions about how aspects of visuomotor performance are influenced by these evaluations. Our framework seeks to overcome these limitations, and address the functional mechanisms by which different responses to stress influence attentional control, visuomotor control, and subsequent motor skill performance.

**FIGURE 1:**
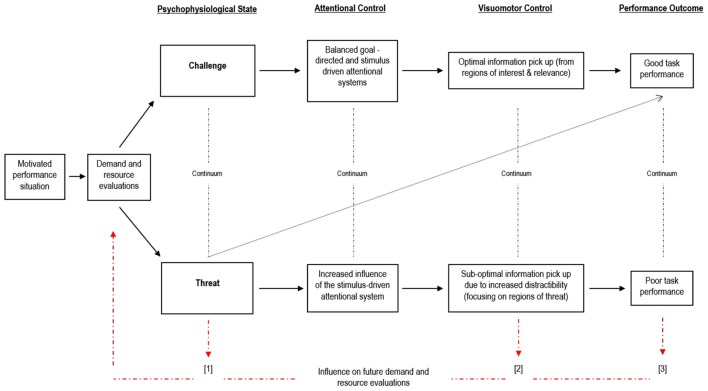
**An integrative framework of stress, attention, and human performance.** The rectangular boxes highlight the opposing psychophysiological responses to stress (challenge or threat state) that result from the demand and resource evaluation process, and their subsequent influence on attentional control and visumomotor performance. The diagonal dashed line represents compensatory strategies (e.g., increasing effort) that can be adopted to prevent a threat state from influencing performance. The red dashed lines represent the cyclical nature of evaluations of stress, and the influence of a threat state on future demand and resource evaluations.

Specifically, in explaining the attentional mechanisms through which stress responses might exert their influence on visuomotor performance, we draw upon the work of Corbetta and colleagues (Corbetta and Shulman, 2002) and Eysenck and colleagues (Attentional Control Theory, ACT; Eysenck et al., 2007). The model of attention articulated by Corbetta et al. (2008) provides an explanation for how attention might be influenced (negatively or positively) under the conditions experienced during a challenge or threat state described by the BPSM. Eysenck et al.’s (2007) ACT highlights the potential role of anxiety in driving the changes to attention and subsequent performance. ACT has been applied to motor skill performance, but is fundamentally interested in the sole effect of anxiety on performance. ACT is weak in explaining the conditions and process by which individuals feel anxious, given that its development was based on comparisons between low and high trait anxious participants (although see Eysenck and Wilson, 2016, for a recent update for sport).

We have also positioned our framework against existing models interested in anxiety, pressure and motor skill performance. A development of ACT, the integrated model of anxiety and perceptual-motor performance (Nieuwenhuys and Oudejans, 2012), is more applicable to motor task performance than ACT and considers state anxiety, but does not explain precisely how competitive pressure influences individuals’ psychophysiological responses to stress and subsequent anxiety levels. Conversely, many existing models of pressure and motor performance such as the explicit monitoring hypothesis (Beilock and Carr, 2001) and the theory of reinvestment (Masters and Maxwell, 2008) consider performance pressure and anxiety, but lack explanatory power in terms of (1) how pressure and stress are interpreted to positively or negatively influence performance, and (2) the precise functions of attentional control that disrupt attention and are relevant to motor skill performance.

We argue that it is the influence of the psychophysiological states (challenge and threat) arising from the stress evaluation process on attentional control, and ultimately visuomotor control, that leads to differential performance outcomes in visuomotor tasks. Finally, we propose empirically driven interventions that might help individuals to perform visuomotor tasks better under stressful conditions, and outline future areas for research enquiry.

## Stress – it is All a Matter of Interpretation

Stress is considered as one of the principle causes of human performance failure. Stress arises when individuals perceive that they cannot adequately cope with the demands being placed on them or with threats to their well-being (Lazarus, 1966). There are clear inter-individual and intra-individual differences in the way that we react to stress (Lazarus, 2000), and a subjective interpretation of stress likely explains this. As such, stress is best conceptualized as a process involving a dynamic interaction between the person and the environment (Lazarus, 1999).

### The Transactional Theory of Stress and Coping

Lazarus and Folkman’s (1984) seminal work on stress describes how humans constantly evaluate what is happening to them, a process known as cognitive appraisal. Cognitive appraisal involves determining the extent to which environmental stressors are harmful, threatening, or challenging (Lazarus, 1966). The process of cognitively appraising harm, threat, and challenge happens in two stages. First, in primary appraisal, the person evaluates whether he or she has anything at stake in this encounter. In the context of performance, social evaluation, monetary incentives, or collective goals may be at stake. Next, in secondary appraisals the person evaluates what, if anything, can be done to overcome these demands, prevent harm, or to improve the prospects for benefit. As such, Lazarus and Folkman (1984) suggested that it is the interpretation of the environment that people face, and the perception of their ability to cope that is critical, rather than the actual environment or actual coping capability. The two-stage cognitive appraisal process is proposed to result in two polarized forms of stress, positive and negative.

Contemporary theories have built upon Lazarus and Folkman’s transactional perspective of stress. For example, Blascovich (2008) proposed the BPSM of challenge and threat. The BPSM re-conceptualized the two-stage appraisal process described by Lazarus and Folkman as a demand (i.e., primary appraisal) and resource (i.e., secondary appraisal) evaluation process. Blascovich (2008) use the term ‘evaluation’ rather than the label ‘appraisal,’ as they deemed it to better reflect the predominately unconscious and automatic (rather than conscious and deliberate) manner in which individuals respond to stress. The BPSM further extended the work of Lazarus and Folkman by linking this evaluation process with the patterns of physiological toughness and weakness outlined by Dienstbier (1989). Using an animal model, Dienstbier noted two patterns of neuroendocrine and cardiovascular responses during stressful performance situations: one among animals who thrived during these situations (termed physiological toughness), and another for animals who did not (termed physiological weakness).

## Biopsychosocial Model of Challenge and Threat

Over the last 20 years, the BPSM has become an increasingly influential theoretical framework to explain individuals’ reactions to stress (Blascovich, 2008). The BPSM contends that how individuals perform in motivated performance situations is determined by a series of psychological evaluations that lead to distinct patterns of physiological responses (Seery, 2013). According to the BPSM, how individuals respond to a stressful situation is determined by their evaluations of situational demands and personal coping resources (Blascovich, 2008). Individuals, who believe that they have sufficient resources to cope with the demands of a situation, *evaluate the situation as a challenge*. Conversely, individuals who judge that they have insufficient coping resources, *evaluate the situation as a threat* (Seery, 2011). The model is designed to explain reactions to situations when performance results are meaningful and task engagement is high; extreme evaluations such as when coping resources grossly exceed task demands (i.e., little or no probability of failure) or vice versa, are predicted to result in disengagement because the task loses its meaning, value, or goal relevance (Blascovich, 2008). As such, the model does not directly consider the impact of boredom or apathy on performance.

The demand and resource evaluation process that leads to a challenge or threat evaluation is dynamic, and is predicted to be influenced by a range of interrelated antecedents including danger, familiarity, uncertainty, required effort, skills, knowledge and abilities, and availability of support (Blascovich, 2008). Despite their discrete labels, it should be noted that challenge and threat evaluations are not viewed as a dichotomy, but rather as anchors of a single bipolar continuum (Seery, 2011). As such, someone can be considered to be more or less challenged, rather than distinctly challenged *or* threatened. The BPSM fails to specify how individuals move along the challenge and threat continuum, and future research is needed to better understand this. Much of the research interested in challenge and threat makes reference to both challenge and threat *states* and challenge and threat *evaluations*. The term *evaluation* reflects the fact that a position on the challenge and threat continuum is the result of a demand and resource evaluation process. The term *state*, is reflective of the psychophysiological state that follows from this evaluation process (outlined below). As such, throughout this review paper we use the terms challenge and/or threat *evaluations* and challenge and/or threat *states* interchangeably.

The BPSM proposes that the demand and resource evaluation process culminates in the triggering of distinct neuroendocrine and cardiovascular responses (Blascovich, 2008). Both challenge and threat evaluations are hypothesized to increase sympathetic-adrenomedullary activation. This activation releases catecholamines (epinephrine and norepinephrine), which cause vasodilation of the blood vessels and higher cardiac activity, resulting in increased blood flow to the brain and muscles. Crucially, a threat evaluation also prompts pituitary-adrenocortical activation. This dampens sympathetic-adrenomedullary activation and releases cortisol, which reduces vasodilation (or causes vasoconstriction) and cardiac activity, resulting in decreased blood flow. A challenge psychophysiological state is therefore marked by relatively higher cardiac output and lower total peripheral resistance compared to a threat psychophysiological state.

Despite the BPSM proposing that different evaluations cause divergent cardiovascular responses, limited research has tested this assumption. In a seminal paper on this topic, Tomaka et al. (1997) found that elicitation of challenge and threat evaluations through instructional sets created a subsequent change in the cardiovascular system in line with the predictions of the BPSM. That is, creating a challenge evaluation led to greater cardiac activity (i.e., heart rate and cardiac output) and less vascular resistance (i.e., total peripheral resistance). More recently, Zanstra et al. (2010) showed that before a stressful presentation, a challenge evaluation was associated with relatively greater decreases in total peripheral resistance and increases in cardiac output. However, some studies have failed to support this assertion. For example, Turner et al. (2012, 2013) found no correlation between self-reported demand and resource evaluations and the cardiovascular markers of challenge and threat states in cricket batting and netball motor tasks, respectively. Similarly, Vine et al. (2013) found the same pattern of results in a surgical task, with subjective self-report and objective cardiovascular markers of challenge and threat states revealing no association. The lack of correlation reported in these studies could be due to the fact that the subjective demand and evaluation process is not leading to the specific cardiovascular responses that are predicted by the BPSM. Alternatively, it could be that either the self-report or cardiovascular measures currently used are not sensitive enough. Improvements to recording equipment and the development of new, more sensitive self-report measures developed specifically for sport (e.g., Rossato et al., 2016), could help resolve this situation.

Despite some mixed findings on the relationship between subjective and objective measures of challenge and threat, research has reliably found support for the BPSM’s contention that a challenge evaluation leads to better performance than a threat evaluation. A number of studies have found that individuals who report a challenge evaluation perform better than individuals who report a threat evaluation (Tomaka et al., 1993; Drach-Zahavy and Erez, 2002; Gildea et al., 2007; White, 2008; Feinberg and Aiello, 2010). For example, O’Connor et al. (2010) asked participants to report evaluated demands and resources before performing a complex negotiation task. The results revealed that evaluating the task as a threat was associated with poorer negotiating performance (i.e., lower quality deals; O’Connor et al., 2010). Similar findings have been reported in medical settings (Roberts et al., 2016) and important sporting competitions (Moore et al., 2013). For instance, Moore et al. (2013) asked experienced golfers to report demand and resource evaluations before an important competition. The results showed that evaluating the competition as a challenge predicted better golf performance (Moore et al., 2013).

Research adopting specific patterns of cardiovascular activity that distinguish challenge and threat states, have also supported the BPSM’s predictions regarding performance (Blascovich et al., 2004; Turner et al., 2012). For example, Seery et al. (2010) found that a challenge cardiovascular response to an academic-relevant speech predicted better exam performance during the subsequent term than a threat response. Furthermore, Turner et al. (2013) found that a challenge cardiovascular response predicted superior batting performance among elite cricketers compared to a threat response. Recent studies have moved beyond these correlational designs to assess the impact of challenge and threat states via experimental manipulations (Moore et al., 2012). For instance, Moore et al. (2013) found that golfers who were experimentally manipulated into a challenge state immediately before a pressurized golf putting task outperformed golfers who were manipulated into a threat state.

While the evidence supporting the core predictions of the BPSM are compelling, the BPSM is limited as it does not provide a clear mechanistic account for why visuomotor performance is influenced differently by challenge and threat states. Subsequently, in the next section, we use the findings from recent research to argue that challenge and threat evaluations predominately impact performance via their effects on attention.

## Attentional Control

Despite its absence from the BPSM, researchers have proposed that attention may be more effective during a challenge evaluation than a threat evaluation (Blascovich et al., 2004; Jones et al., 2009). Specifically, attention may be focused on task-relevant cues following a challenge evaluation, but toward task-irrelevant (and potentially threatening) cues, or controlling one’s own actions, following a threat evaluation (Blascovich et al., 2004; Jones et al., 2009). Recent research has begun investigating these propositions (Sassenberg et al., 2015). For example, Frings et al. (2014) asked participants to complete a visual search task that involved locating a target appearing in one of two search arrays: one associated with gaining points and another associated with avoiding the loss of points. The results suggested that participants who were manipulated into a challenge evaluation (via mid-task performance feedback), spent more time searching the gain array and made fewer fixations toward the loss array. In contrast, participants manipulated into a threat evaluation made fewer fixations to the gain array. These results suggest that a threat evaluation may be associated with greater detection of negative (or threatening) stimuli. An effect that is likely to maintain or exacerbate a threat evaluation in the future (Frings et al., 2014).

In visuomotor tasks, effective visual attention is critical if the necessary information is to be acquired for the accurate planning and control of movements (e.g., Land, 2009). Contemporary research has demonstrated that challenge and threat evaluations can have divergent effects on attention and subsequent motor control. For example, Moore et al. (2012) manipulated novice golfers into either a challenge or a threat state before a pressurized golf putting task. As well as outperforming participants manipulated into a threat state, the participants who were manipulated into a challenge state fixated the golf ball for longer before initiating the putting action (i.e., longer quiet eye durations; Vickers, 2007), indicating superior task-specific visuomotor control. These results were replicated in a follow-up study, with experienced golfers manipulated into a threat state displaying inferior performance and shorter fixations on the golf ball before the putting action compared to golfers manipulated into a challenge state (Moore et al., 2013).

Similar findings have been reported for other visuomotor tasks. For instance, Vine et al. (2013) asked participants to complete a baseline trial on a novel surgical task, before being trained to proficiency and then re-performing the task under stressful conditions. The authors found that during baseline and stressful trials, evaluating the task as a challenge was associated with superior motor performance (i.e., quicker completion times) and attentional control (i.e., fixating the target and ignoring other distracting information in the environment; target-locking). Vine et al. (2015) also examined pilots’ stress responses, attentional control, and performance during a critical incident (engine failure on takeoff) during mandatory license check assessments in a simulator. They found that evaluating the task as a threat predicted poorer performance (i.e., lower instructor evaluations, greater heading, and speed deviations) and disrupted attentional control (i.e., fixating more areas of the cockpit and spending more time fixating task-irrelevant areas; Vine et al., 2015).

Despite there being compelling evidence to support the influence of challenge and threat states on attentional control, there is a lack of a systematic explanation of these effects. It is this gap in current understanding that this integrative framework intends to fill. In systematically explaining the aforementioned attentional differences between challenge and threat states, we refer to a seminal model of attentional control proposed by Corbetta and Shulman (2002). These authors described goal-directed and stimulus-driven attentional systems that serve distinct, yet complimentary, roles in the control of attention. The top-down (goal-directed) control system is centered on the dorsal posterior parietal and frontal cortex, and is involved in preparing and applying goal-directed selection of stimuli and action responses. In contrast, the stimulus-driven control system includes the temporoparietal cortex and inferior frontal cortex, and is largely lateralized to the right hemisphere (Corbetta and Shulman, 2002). This system acts as a “circuit breaker” (2002, p. 201) for the dorsal system, and in normal functioning both systems work together so that attention can be flexibly allocated.

Differences in the gaze behaviors displayed during challenge and threat evaluations could be explained by imbalances in these attentional systems. Indeed, longer quiet eye durations (Moore et al., 2012, 2013); greater target locking (Vine et al., 2013); less distractibility (Vine et al., 2015); and a bias toward gains rather than losses (Frings et al., 2014), have all been adopted to explain attentional differences between challenge and threat states, and likely reflect a maintenance of the goal-directed attention system. Thus, when a challenge evaluation ensues, the goal-directed and stimulus-driven attentional systems are balanced, resulting in sustained attention and optimal information processing from task-relevant areas. In contrast, when threat evaluation ensues, the stimulus-driven attentional system dominates the goal-directed attentional system, resulting in greater distractibility by task-irrelevant (and/or threatening) stimuli and sub-optimal processing of task-relevant information (as reflected by shorter quiet eye durations, less target locking, greater distractibility, and a bias toward losses). The proposed integrative framework (**Figure 1**) highlights the fundamental differences in attentional control between challenge and threat evaluations (i.e., goal-directed vs. stimulus-driven attention) and the resultant differences in visuomotor performance.

## How Does a Threat Evaluation Lead to Disrupted Attention?

What is less clear within the current literature are the precise mechanisms through which challenge and threat evaluations lead to differences in attentional control, or more specifically, why a threat evaluation leads to disrupted attention. There are several potential mechanisms that warrant future research. For example, challenge and threat states have been associated with differential motivational orientations. While a challenge state has been associated with an approach motivational orientation, a threat state has been associated with an avoidance motivational orientation (see Turner et al., 2013)^1^. Approach motivation (also referred to as promotion focus) involves an individual working toward a desirable end goal (i.e., winning a game, or achieving a medal), whereas avoidance motivation (also known as prevention focus) involves someone trying to avoid undesirable end states (i.e., avoiding being last or making a mistake; see Maddox et al., 2010). Interestingly, these motivational states have been associated with differences in decision making and response selection (Markman et al., 2005), as well as attentional flexibility (Calcott and Berkman, 2014). As such, it may be the avoidance orientation adopted by an individual experiencing a threat state that leads to disrupted attention (see Jones et al., 2009).

It is also possible that the cardiovascular differences between challenge and threat states explain the differences in attentional control. For example, Jamieson et al. (2012) showed that a more adaptive physiological response (akin to a challenge state) exhibited reduced threat-related attentional bias. Future research should also consider the influence of arousal, which has been shown to effect cognitive functions relating to attention (Humphreys and Revelle, 1984), and aspects of information processing and perception (Sanders, 1983). Similarly, the neuroendocrine responses accompanying challenge and threat states may also influence aspects of cognition relating to attention. For example, cortisol (which is higher in a threat state than a challenge state) has been associated with greater vigilance to threatening stimuli under stressful conditions (see Akinola and Mendes, 2012). However, based on existing research, the most likely candidate is the emotion of anxiety.

## The Role of Anxiety

Researchers have suggested that challenge and threat evaluations may lead to different emotional responses (Blascovich, 2008; Jones et al., 2009). While correlational research has revealed little or no relationship between a threat evaluation and increased cognitive anxiety (Turner et al., 2012, 2013; Meijen et al., 2013a,b), experimental research has supported this assumption and has demonstrated that a threat evaluation is linked with higher levels of cognitive anxiety than a challenge evaluation (Williams et al., 2010; Williams and Cumming, 2012; Moore et al., 2013). For example, Moore et al. (2012) found that participants who were verbally manipulated into a threat state reported experiencing greater cognitive anxiety than participants who were manipulated into a challenge state. Furthermore, participants in the threat group also reported experiencing greater somatic anxiety and interpreted cognitive and somatic anxiety symptoms as more debilitative for their performance than participants in the challenge group (Moore et al., 2012).

Given that a threat evaluation is associated with heightened levels of cognitive anxiety, and the nature of the subsequent changes in attentional control, parallels can be drawn to the predictions of ACT (Eysenck et al., 2007), helping explain why challenge and threat states have differential effects on attention and performance. According to ACT, anxiety disrupts attention, diverting processing resources from task-relevant stimuli to task-irrelevant (and often threatening) stimuli. The effects of anxiety on attention are said to occur for both external (e.g., environmental distractors) and internal (e.g., negative thoughts, body sensations) stimuli. Eysenck et al. (2007) proposed that this impairment in attentional control occurs due to an imbalance between the two attentional systems outlined by Corbetta et al. (2008). Anxiety is predicted to increase the sensitivity of the stimulus-driven system at the expense of the goal-directed system, making individuals more distractible and less able to maintain focused, goal-orientated attention (Eysenck et al., 2007). As such, if anxiety increases with a threat evaluation (and decreases with a challenge evaluation) then this might explain why a threat state is associated with disrupted attention. Our interpretation of how pressure influences attention is not at odds with existing explanations of pressure and motor skill performance. Indeed, changes in the balance of the stimulus driven and goal directed attentional systems might account for the monitoring of movement effects described in self-focus accounts of choking (e.g., Masters, 1992; Beilock and Carr, 2001), as attention may become inefficiently pulled toward salient but disruptive movement cues when anxious (see also Eysenck and Wilson, 2016). The framework we propose is more specific regarding the precise functions of attention that may cause these disruptions, and future research should further examine this contention to ‘bridge the gap’ between self-focus and distraction theories of pressure and performance.

While a complete review of the predictions of ACT is beyond the scope of this article (see Eysenck and Wilson, 2016 for a recent review and update), it is important to note that considerable research has supported the attentional disruptions proposed by ACT in visuomotor tasks, and many of these studies have adopted similar measures of attention to those studies interested in comparing challenge and threat states (Nieuwenhuys et al., 2008; Wilson et al., 2009b; Niewenhuys and Oudejans, 2010; Causer et al., 2011; Nibbeling et al., 2012; Allsop and Gray, 2013). For example, Allsop and Gray (2014) examined the influence of anxiety on gaze behavior in a simulated aviation task. They found that under anxious conditions, participants spent more time fixating the outside world and displayed more random visual search (i.e., entropy), suggesting an increased influence of the stimulus-driven system (Allsop and Gray, 2014). Furthermore, in sport, Wilson et al. (2009a) investigated the effects of anxiety on gaze behavior in a basketball free throw task. They found that under elevated anxiety, participants spent less time fixating the target before initiating each free throw (i.e., shorter quiet eye durations), reflecting impaired goal-directed attentional control. Additionally, participants displayed more fixations of a shorter duration to various locations (i.e., higher visual search rate), suggesting an increased influence of the stimulus-driven system (Wilson et al., 2009a).

While both the BPSM and ACT were not originally developed to explain performance variability in visuomotor tasks performed under pressure, both accounts have received support in this field. However, limitations in both accounts provide the opportunity to develop an integrative model that might better explain the experience of stress in sport (and other evaluative settings) and its impact on visuomotor performance. For example, while the BPSM’s strength lies in its transactional approach and its ability to categorize individuals based on their different responses to stress, it is weak in explaining how these changes will influence visuomotor control and sporting performance (see also Jones et al., 2009) ^2^. ACT, through its focus on attentional disruptions, provides a mechanistic explanation for how negative stress responses might functionally disrupt the processing of critical information that is used to plan and control visually guided movements. However, it is weak in explaining precisely how pressure influences individuals psychophysiological responses to stress and subsequent anxiety levels, a limitation that is also inherent in other models of pressure and perceptual-motor performance such as the explicit monitoring hypothesis (Beilock and Carr, 2001), theory of reinvestment (Masters and Maxwell, 2008), and integrated model of anxiety and perceptual-motor performance (Nieuwenhuys and Oudejans, 2012). Not only does this integrative framework functionally align these limitations and strengths, but importantly, it also proposes stages at which interventions might be introduced to improve or support performance.

## Integrative Conceptual Framework

Based on the findings (and limitations) of the aforementioned research, we propose an integrative framework to offer an evidence-based explanation of performance variability under stress (**Figure 1**). This framework is supported by the findings of the research outlined above. Consistent with the BPSM, this integrative framework applies to pressurized situations in which individuals are required to perform visuomotor tasks to attain an important and meaningful goal. According to this framework, individuals actively engaging in a stressful situation consciously and subconsciously evaluate the demands of the situation and their ability to cope with these demands. Individuals who judge that they have sufficient resources to cope with the demands of the situation, evaluate the situation as a challenge. In contrast, individuals who believe that they do not possess the resources required to cope with the demands of the situation, evaluate the situation as a threat. Congruent with the BPSM, the integrative model proposes that a challenge evaluation will lead to a cardiovascular response consisting of relatively higher cardiac output and lower total peripheral resistance compared to a threat evaluation. These cardiovascular indices can be used to determine underlying demand and resource evaluations in an objective and online manner, avoiding the issues associated with self-report measures (e.g., social desirability bias).

Next, this integrative framework purports that challenge and threat evaluations have different effects on attentional control. A challenge evaluation is characterized by a balanced influence of goal-directed and stimulus-driven attention systems, in contrast to a threat evaluation which is characterized by an increased influence of the stimulus-driven attentional system. We propose several potential mechanisms through which this effect occurs (i.e., motivational orientation, cardiovascular and endocrine responses), but emphasize the likely important role of the increase in cognitive anxiety associated with a threat evaluation. Importantly for visuomotor performance, the increased influence of bottom–up (stimulus-driven) attentional control, can lead to increased distractibility (particularly by threatening and salient stimuli), which translates into a threat evaluation being associated with disrupted visuomotor control. In this sense, an individual with a threat evaluation will not exert the necessary attentional control to enable them to pick-up all of the relevant information needed to accurately perform a motor skill, and may have a tendency to focus on regions of threat or other irrelevant sources (Wilson et al., 2009b). Conversely, an individual with a challenge evaluation will be able to effectively control attention, and focus on regions of importance, therefore picking up the optimal visual information needed to perform the motor skill accurately. Ultimately, as a result of these differences in visuomotor control, task performance is likely to differ; for a given performance potential, individuals in a challenge state will be better able to perform skills than individuals in a threat state.

However, taking from a key tenet of ACT, we propose that a threat evaluation may not always influence the overall effectiveness of performance (Eysenck et al., 2007). Despite having impaired physiological functioning and attentional control it is possible to maintain performance, if additional processing resources (effort) are mobilized. This is important because it reflects that, while at a group level a challenge evaluation is associated with better performance, it is still possible for an individual to perform well even adopting a threat evaluation (and vice versa). For example, Turner et al. (2013) reported that some individuals who displayed a challenge cardiovascular response still performed poorly (in comparison to those who displayed a threat cardiovascular response). Future research should examine the role of effort in such instances. In contrast to ACT however, we predict that this mobilization of effort may be in response to the initial demand and resource evaluation process (i.e., demands perceived to outweigh resources) rather than in response to anxiety alone (as proposed by ACT). Blascovich (2008) proposed effort to be an important antecedent of challenge and threat states, and recent experimental work has supported this contention (Moore et al., 2014). Moore et al. (2014) found that performers of a surgical motor task were sensitive to the amount of effort required to perform the task. Perceptions of high effort led to a threat state in comparison to perceptions of low effort, which led to a challenge state. Future work should attempt to understand precisely when perceptions of effort are influential, and the effect that the mobilization of effort has on performance outcomes.

There may also be occasions in which a threat evaluation is beneficial for performance because of the specific demands of the task being performed. For example, there is evidence that for a task that requires vigilance, a threat state is better than a challenge state (Blascovich, 2008). These compensatory processes of effort, and the paradoxical effect of adopting a threat evaluation but performing well, are indicated on the model by the dashed diagonal line from a threat evaluation to good task performance. These contentions warrant further investigation.

## Feedback Loops and Interventions

We propose three feedback loops within our framework that highlight how an individual’s reaction to stress may be self-perpetuating. As such, these feedback loops also highlight the potential for targeted interventions that are designed to help performers maintain or improve performance in pressurized environments. The first feedback loop (1) arises from the psychophysiological state experienced by the performer. It is likely that the physiological and emotional consequences of a threat evaluation (i.e., heightened arousal and anxiety) will further increase the likelihood that the individual will subsequently evaluate the task as highly demanding, and judge that they have fewer coping resources, making a subsequent threat evaluation more likely. In essence, the preceding threat state becomes an extra processing demand for the individual to evaluate. Interventions that are designed to help athletes to re-frame these symptoms may be a useful way to ‘break the cycle’ and help an individual to evaluate a situation as a challenge (see Jamieson et al., 2012). Indeed, a recent study by Moore et al. (2015) showed that an arousal re-appraisal intervention helped individuals to overcome a threat state, and subsequently adopt a challenge state, leading to better performance on a pressurized golf-putting task. Furthermore, Brooks (2014) revealed that interpreting anxiety as excitement (a cognitive re-appraisal), led to improved performance outcomes in a number of tasks. The re-appraisal of a high arousal emotion (anxiety) as another high-arousal emotion (excitement) may therefore be effective in reducing the negative effects of anxiety on motor task performance. We propose that such interventions will influence subsequent demand and resource evaluations positively and protect visuomotor performance in the long term.

The second feedback loop (2) arises from the differences in attentional control and visuomotor control experienced as a result of challenge and threat evaluations. Following a threat evaluation, individuals are unable to maintain effective top down attentional control, and tend to focus on task-irrelevant (and often threatening) stimuli due to distractibility. We argue that this will likely serve to further skew their demand and resource evaluations and further reinforce a threat evaluation (Yiend, 2010). For example, the anxiety experienced by individuals in a threat state will likely lead to an increase focus on sources of threat, and a tendency to reach a pessimistic interpretation of ambiguous events (MacLeod and Mathews, 1988; Mathews and Mackintosh, 1998). Similarly, anxiety is suggested to cause a threat related interpretation of information, and a tendency to display emotion-congruent behavior (Nieuwenhuys and Oudejans, 2012). Collectively, this might act as a self-perpetuating cycle that negatively influences the demand and resource evaluation process, resulting in threat evaluations.

As such, we are proposing that the control of attention is critical in the demand and resource evaluation process, giving individuals the capability to accurately perceive the demands and resources relevant to the situation. Studies are beginning to investigate whether training attention away from threat (negative visual cues) can reduce this self-perpetuating effect and reduce the symptoms of anxiety (Amir et al., 2008; Schmidt et al., 2009). Furthermore, Nieuwenhuys and Oudejans (2011) have shown that training with anxiety can help to overcome the detrimental effect of anxiety on attention and help performance maintenance (see also Oudejans and Pijpers, 2009, 2010). Training with anxiety might therefore lead to future situations being evaluated as less demanding and/or the individual evaluating that they have sufficient coping resources, due to the previous stressful situations they have encountered.

The third feedback loop (3) is related to the effects of performance outcome on the subsequent demand and resource evaluation process. In this integrative framework we argue that good task performance will lead individuals to evaluate similar tasks in the future as less demanding and to judge that they have sufficient resources to cope with these demands (i.e., a challenge evaluation). In contrast, we believe that poor performance will cause individuals to evaluate comparable tasks as more demanding and to evaluate that they lack the necessary resources to cope with these demands (i.e., a threat evaluation). Essentially, we propose that successful previous performances become a future resource, while unsuccessful prior performances become a future demand. Limited research has tested this assertion (Quigley et al., 2002). However, Rith-Najarian et al. (2014) reported that performance on a speech task predicted subsequent demand and resource evaluations, with poorer performance associated with a threat evaluation. Future studies should test this feedback loop using within-subjects designs and visuomotor tasks to add to our understanding of the underlying mechanisms.

## Future Directions and Concluding Remarks

Although, recent research has offered support for much of what we have articulated in this article, future research is encouraged to further scrutinize the findings discussed and further test and expand the framework. In addition to the suggestions in the previous section, a number of potential avenues for future research exist. For instance, the BPSM suggests a number of interrelated factors that might influence the demand and resource evaluation process, and lead to a challenge or threat evaluation, including danger, familiarity, uncertainty, required effort, skills, knowledge and abilities, and availability of support (Blascovich, 2008). To date, only one study has experimentally examined these antecedents (Moore et al., 2014). These authors found that while perceptions of required effort influenced demand and resource evaluations, cardiovascular responses, and performance during a novel motor task, perceptions of support availability had little effect. Additional work is needed to explore the complex interplay between these antecedents and how they collectively influence challenge and threat states and visuomotor performance. It may also be fruitful to examine if the antecedents proposed by other theories (e.g., TCTSA; see Jones et al., 2009) and key dispositions (e.g., hardiness; see Eschleman et al., 2010) influence the relationships outlined in the framework.

Second, more research is needed to understand precisely why attention is disrupted following a threat evaluation. In the current paper, we have made the case for anxiety being the critical emotion that distinguishes between challenge and threat states, causing disruptions to attention. However, as we have also proposed, it is possible that attentional disruptions that result from a threat evaluation might be due to other factors (e.g., motivational orientation; neuroendocrine and cardiovascular responses). Finally, future research might consider a more fine-grained examination of the continuum between challenge and threat evaluations that we propose in our framework. Better categorization of the differences between challenge and threat evaluations might lead to a more detailed understanding of the attentional and visuomotor changes that influence performance. For example, researchers should consider differences in individuals who evaluate resources that significantly outweigh demands (i.e., high challenge); those that evaluate resources that only just outweigh or match demands (i.e., low challenge); those that evaluate resources that are just shy of demands (i.e., low threat); and those that evaluate resources that are completely insufficient (i.e., high threat). It is also imperative for research to further explore individuals that do not fit with the predictions of the framework (e.g., individuals in a threat state who perform well), and consider explanatory factors (e.g., emotions, motivation, and effort).

To conclude, this article proposes a novel integrative framework in the light of contemporary research that might help explain the effects of stress on attention and visuomotor performance. It is hoped that this framework will provide researchers with a novel perspective of stress and visuomotor performance, based on a synthesis of existing literature. We also hope that it will provide researchers with testable hypotheses for future research, and practitioners with a practical tool to understand and improve performance under pressure.

## Author Contributions

SV, LM, and MW contributed to the development of the ideas and the write up of the paper.

## Conflict of Interest Statement

The authors declare that the research was conducted in the absence of any commercial or financial relationships that could be construed as a potential conflict of interest.

## Footnotes

1 For a comprehensive discussion of the proposed differential emotional reactions to challenge and threat evaluations see Jones et al. (2009).

2 Jones et al. (2009) applied the principles of the BPSM to sporting performance in their theory of challenge and threat states in athletes (TCTSA).
